# S11 How to increase the reach of HEPA promotion among socially disadvantaged groups?

**DOI:** 10.1093/eurpub/ckae114.245

**Published:** 2024-09-26

**Authors:** Niamh Murphy

**Affiliations:** South East Technological University (SETU), Ireland

## Abstract

Participation in physical activity (PA) has many benefits beyond improvements in physical health, like improved mental health, social connections, and certain skills. People from socially disadvantaged groups may benefit most from these outcomes, yet, programs that aim to increase physical activity participation among such groups often struggle with reaching and engaging adequate numbers of participants. This is unfortunate, because no matter how effective a program is for improving health and PA participation, its success and continuation is largely determined by its initial reach. In this symposium, chaired by Niamh Murphy from SETU Waterford in Ireland, we present recent insights obtained through research and practice into factors that affect the reach of physical activity interventions for various marginalized groups.

Maike Till, Karim Abu-Omar, and Heiko Ziemainz from Friedrich-Alexander-Universität Erlangen-Nürnberg, Germany, discuss findings of the community-based participatory research project “BIG”, which explored how women in difficult life situations can best be reached and encouraged to participate in physical activity. The results showed, among others, that this may require distinct approaches for women in urban and rural areas.

Jennifer Thomas, Diane Crone, Nic Bowes, Katie Thirlaway, Robert Meyers, and Kelly Mackintosh from Swansea University in Wales, UK, studied the barriers and opportunities for reaching and recruiting young people experiencing homelessness. They will present the most important factors that they identified at organizational, individual and intervention levels.

Kirsten Verkooijen, Daniëlla van Uden, Güven Alarslan, Dico de Jager and Annemarie Wagemakers from Wageningen University and Research in the Netherlands will present the most important lessons regarding program reach, based on their experience with multiple physical activity programs that are specifically designed for socially disadvantaged groups.

Finally, Seamus Nugent, a sports inclusion development officer of the Kilkenny Recreation and Sports Partnership in Ireland, will bring in a practice perspective on reaching, and providing inclusive, meaningful physical activity opportunities for children and adults living with disability in Ireland.

